# MicroRNA-275 and its target Vitellogenin-2 are crucial in ovary development and blood digestion of *Haemaphysalis longicornis*

**DOI:** 10.1186/s13071-017-2153-1

**Published:** 2017-05-22

**Authors:** Jiawei Hao, Jin Luo, Ze Chen, Qiaoyun Ren, Jinxia Guo, Xiaocui Liu, Qiuyu Chen, Feng Wu, Zhen Wang, Jianxun Luo, Hong Yin, Hui Wang, Guangyuan Liu

**Affiliations:** 1 State Key Laboratory of Veterinary Etiological Biology, Key Laboratory of Veterinary Parasitology of Gansu Province, Lanzhou Veterinary Research Institute, Chinese Academy of Agricultural Science, Xujiaping 1, Lanzhou, Gansu 730046 People’s Republic of China; 2 Jiangsu Co-innovation Center for Prevention and Control of Important Animal Infectious Diseases and Zoonoses, Yangzhou, 225009 People’s Republic of China; 3 NERC/Centre for Ecology and Hydrology (CEH) Wallingford, Crowmarsh Gifford, Wallingford, Oxon OX10 8BB UK; 4 Institute of Biomedical Engineering (IBME), Department of Engineering, University of Oxford, Oxford, OX3 7DQ UK

**Keywords:** *Haemaphysalis longicornis*, MicroRNA, Vitellogenin, Blood digestion, Ovary development

## Abstract

**Background:**

The hard tick *Haemaphysalis longicornis* is widely distributed in eastern Asia, New Zealand and Australia and is considered the major vector of *Theileria* and *Babesia*, harmful parasites to humans and animals. Female ticks need successful blood meals to complete the life-cycle. Therefore, elucidation of the underlying molecular mechanisms of *H. longicornis* development and reproduction is considered important for developing control strategies against the tick and tick-borne pathogens.

**Methods:**

Luciferase assays were used to identify the targets of micro RNA miR-275 *in vitro*. RNAi of Vitellogenin (Vg) was used in phenotype rescue experiments of ticks with miR-275 inhibition, and these analyses were used to identify the authentic target of miR-275 *in vivo*. The expression of miR-275 in different tissues and developmental stages of ticks was assessed by real-time PCR. To elucidate the functions of miR-275 in female ticks, we injected a miR-275 antagomir into female ticks and observed the phenotypic changes. Statistical analyses were performed with GraphPad5 using Student’s *t*-test.

**Results:**

In this study, we identified Vg-2 as an authentic target of miR-275 both *in vitro* and *in vivo* by luciferase assays and phenotype rescue experiments. miR-275 plays the regulatory role in a tissue-specific manner and differentially in developmental stages. Silencing of miR-275 resulted in blood digestion problems, substantially impaired ovary development and significantly reduced egg mass (*P* < 0.0001). Furthermore, RNAi silencing of Vg-2 not only impacted the blood meal uptake (*P* < 0.05) but also the egg mass (*P* < 0.05). Significant rescue was observed in miR-275 knockout ticks when RNAi was applied to Vg-2.

**Conclusion:**

To our knowledge, this study is the first demonstration that miR-275 targets Vg-2 in *H. longicornis* and regulates the functions of blood digestion and ovary development. These findings improve the molecular understanding of tick development and reproduction.

## Background

Ticks are hematophagous ectoparasites that require blood meals from vertebrates to obtain a variety of nutrients. They transmit many vector-borne pathogens to humans and animals [1] and cause significant economic losses [2, 3]. Ticks are considered one the most common vectors of human and animal diseases worldwide, second only to mosquitoes [4]. The hard tick *Haemaphysalis longicornis* is found in eastern Asia, New Zealand and Australia and is distributed in most regions of China. This species carries *Theileria* spp. and *Babesia* spp., which are harmful parasites in humans and animals [5, 6].

MicroRNAs (miRNAs) are the most abundant class of small non-coding RNAs. They are 19–24 nt in length and regulate gene expressions at the post-transcriptional level by binding to target mRNAs [7, 8]. In animals, miRNAs recognize their target sites by incomplete base pairing [9], and nucleotides 2–7 (the seed region) are complementary to the target mRNA. Most miRNAs act on 3' untranslated regions (3' UTR) of the target mRNA [10], but they can also bind to the 5' UTR [11, 12] and the open reading frame (ORF) [13, 14].

Vitellogenin (Vg), common to oviparous animals, is a class of phospho-lipoglycoprotein and is the precursor of Vitellin [15]. In ticks, Vg is predominantly synthesized in the fat body and regulated by 20-hydroxyecdysone; it is transported to specific tissues after a blood meal [16, 17]. Vg provides amino acids, carbohydrates and other nutrients for embryonic development. Vg is not only found in fed adults but also in larvae and eggs [16]. Multiple Vgs were previously detected in different tissues of *H. longicornis* [15]. Vg could regulate the expression of microRNAs and may affect foraging behavior in honey bees [18]. In zebrafish, miRNA expression was also associated with Vgs, and six differentially expressed miRNAs may potentially interact with Vgs [19]. Although several tick specific-miRNAs have been identified in different developmental stages [20], thus far, *in vivo* functional studies are lacking. A previous study demonstrated that miRNA-275 is crucial for blood digestion and egg development in *Aedes aegypti* [21]. miR-275 is one of the 10 most abundant miRNAs in female *Rhipicephalus haemaphysaloides* ticks, but not in males, indicating that miR-275 has a critical role in females [22]. However, the function of miRNA-275 in ticks is unclear. Thus, to determine the roles of miRNA-275 and Vg in *H. longicornis* ticks, we identified the putative miR-275 binding sites within Vg-2 and examined the role of miR-275 in regulating ovarian development and blood digestion.

## Methods

### Tick rearing

*Haemaphysalis longicornis* ticks were collected from the Gansu Province and have been maintained in our laboratory since 2006. Adult ticks fed on New Zealand white rabbits. After detachment, the ticks were reared at 28 ± 1 °C and 75% humidity in a drying basin.

### Computational prediction of miRNA targets

To predict putative gene targets of miR-275 (MI0012273), we used the RNAhybrid programme to identify miR-275 binding sites in the complete sequence of Vgs. There are four known *H. longicornis* Vgs (HlVg-1, HlVg-2, HlVg-3 and HlVg-B) available in GenBank-NCBI, and we selected HlVg-1, HlVg-2, and HlVg-3 for analysis because the specific functions of HlVg-B are unknown.

### Cell culture and luciferase assay

The 293 T cell line was used in this study. Cells were maintained in Dulbecco's modified Eagle’s medium (DMEM) (Gibco, Waltham, USA) supplemented with 10% fetal bovine serum (Gibco), penicillin and streptomycin and maintained in an incubator with 5% CO_2_ at 37 °C. The predicted binding sites were cloned and then inserted into the pmirGLO (Promega, Madison, USA) reporter vector. For reporter assays, 150 ng of pmirGLO reporter vector and 50 nM miR-275 mimic (RiboBio, GuangZhou, China) were co-transfected into 293 T cells using Lipofectamine 2000. No-mimic treatment cells were used as a blank control, and pmirGLO-Vg vector only cells were used as the negative control. *Firefly* and *Renilla* luciferase activities were measured 48 h post-transfection by the Dual-Luciferase Reporter Assay System (Promega). First, 100 μl of luciferase assay reagent II was added to each well, *Firefly* luciferase activities was measured, and 100 μl Stop&Glo reagent was loaded into each well. Then, *Renilla* luciferase activities were measured. *Firefly* luciferase in the pmirGLO vector was used for normalization of *Renilla* luciferase expression. Treatments were assessed in triplicate, and transfections were repeated three times. *Firefly* luciferase activities were divided by *Renilla* luciferase activities. Finally, the ratio between *Renilla* and *Firefly* luciferase activities was calculated for each experiment.

### Synthesis and application of antagomir

Antagomirs are miRNA-specific antisense oligonucleotides and were synthetized by Dharmacon (http://dharmacon.gelifesciences.com). The miRNA-275 antagomir (Ant-275) was the reverse complement of mature miRNA-275, and chemical modification was performed as described in a previous study [23] (5'-mC.*.mG.*.mC.mG.mC.mG.mC.mU.mA.mC.mU.mU.mC.mA.mG.mG.mU.mA.mC.mC.*.mU.*.mG.*.mA.*-Chl-3'). The “missense” (MsAnt) sequence (5'-mC.*.mG.*.mC.mU.mU.mU.mC.mG.mU.mG.mG.mU.mU.mC.mU.mG.mG.mU.mA.mC.*.mC.*.mU.*.mU.*-Chl-3') was used as the negative control of the antagomir. [“*” is a phosphate backbone modification that was introduced to increase nuclease resistance and facilitate cellular uptake and bioavailability *in vivo*. “m” is a 2'-O-methyl (2'-OMe) modification, which reduces off-targeting. “Chl” is cholesterol, which can enhance gene silencing *in vivo*]. Non-injected ticks were used as a blank control group. To assess the specificity of the antagomir, we measured the levels of other miRNAs, such as miRNA-10. Antagomirs were microinjected into unfed adult female *H. longicornis* at a dose of 400 μM in 0.5 μl. Every group had 30 female ticks in total, and the groups fed on animals for blood-feeding 24 h after microinjection.

### Phenotype rescue experiment and Vg-2 RNAi

The primers F: 5'-GGA TCC TAA TAC GAC TCA CTA TAG GCT TTG GAG AGT ACT CCA AGA AC-3' and R: 5'-GGA TCC TAA TAC GAC TCA CTA TAG GGG CCG TCG GGC CCG CTC AGG T-3' were used to amplify Vg-2 [15]. Primers for PCR analysis of the plasmid pmirGLO/Luciferase were as follows: F: 5'-GGT ACC TAA TAC GAC TCA CTA TAG GAT CCA GAA CAA AGG AAA CGG-3' and R: 5'-GGT ACC TAA TAC GAC TCA CTA TAG GCC AAA CAA GCA CCC CAA T-3'; the T7 promoters are underlined. We used the T7 RiboMax Express RNAi system (Promega) to synthesize dsRNA based on the manufacturer’s instructions. The dsRNA for luciferase (dsLuc) was used as a negative control. A total of 2 μg dsRNA in 0.5 μl water was injected into each unfed female. In the rescue experiments, the ticks were divided into five groups as follows: non-injection; Vg-dsRNA injection; miR-275 antagomir (Ant-275) injection, co-injection of Ant-275 and dsVg (Ant-275/dsVg); and co-injection of Ant-275 and dsLuc (Ant-275/dsLuc). Every group contained 30 female ticks in total (Table 1). Phenotypic measurements included egg number, engorgement weight, final weight, duration of egg laying and the rate of weight decrease of engorged females. Injected ticks were allowed to recover for 24 h before blood-feeding.

**Table 1:** Phenotype rescue experiment group

Group	Injection dose/μl	No of ticks	Application
Non-inj	0	30	Blank control
Ant-275	400 μM	30	Experimental
dsVg	2 μg	30	Experimental
Ant-275/dsVg	400 μM + 2 μg	30	Experimental
Ant-275/dsLuc	400 μM + 2 μg	30	Negative control

### Total RNA isolation and real-time PCR

Total RNA was extracted from various tissues and development stages of ticks with TRIzol reagent (TaKaRa, Dalian, China) according to the manufacturer’s protocol, and miScript (TaKaRa) was utilized for mRNA and miRNA cDNA synthesis. Real-time PCR was performed to analyze the expression of miRNA and mRNA using a SYBR Prime Script miRNA RT-PCR kit. Specific primers for Vg-2 (forward primer 5'-TGG TGG ACA AGG GAT GAG AA-3' and reverse primer 5'-ATC CGA GCG AAC TGA AGA CC-3'), miRNA-275 (5'-CAG GTA CCT GAA GTA GCG CG-3') and miRNA-10 (5'-ACC CTG TAG ATC CGA ATT TGT-3') were used in this study. U6 and β-actin were also amplified as internal controls. Relative expression of miRNAs and Vg-2 was calculated by the 2^-ΔCt^ method.

### Statistical analysis

All data were analyzed with GraphPad 5 using Student’s *t-*test. Probability values of less than 0.05 were considered significant, and the results are shown as the mean ± SEM.

## Results

### miR-275 targets Vg-2 *in vitro*

**Fig. 1:**
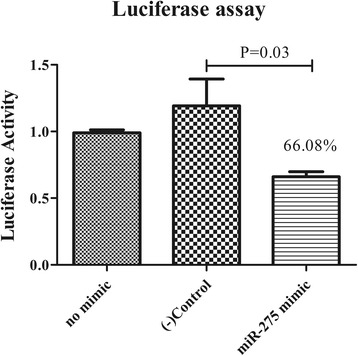
Vg-2 is a target of miR-275. Dual Luciferase reporter assays for Vg-2 are represented as the mean ± SEM of triplicate samples

The putative target genes (Vgs) of miR-275 were identified using RNAhybrid. The complete sequence of Vgs was used to predict the binding sites. Computational analysis showed that there were many potential binding sites in Vgs, but only two predicted results within the 7-mer seed sequence sites (we chose the definition of the canonical seed binding site, no U:G pairing in the seed sequence) in Vg-2 (Table 2). Based on these results, the two binding sites were cloned and inserted downstream of *Renilla* in the pmirGLO vector, which was then co-transfected into 293 T cells with miR-275 mimics. Luciferase reporter assays showed that only one site (position 1461) resulted in 66.07% luciferase activity compared with that of the negative control and no mimic control (Fig. 1) (*t*_(4)_ = 2.599, *P* = 0.03); the other site resulted in no significant difference compared with that of the control. Therefore, the results strongly indicated that Vg-2 is a potential target of miR-275 *in vitro*.

**Table 2:** Putative miR-275 binding sites of Vg-2

miRNA	Position	mfe^a^	Target site
miR-275	1461	-21.9 kcal/mol	
miR-275	1568	-22 kcal/mol	

^a^ Minimum free energy (mfe) values based on RNAhybrid prediction

### Expression analysis of miR-275 and Vg-2 from various developmental stages and tissues in ticks

**Fig. 2:**
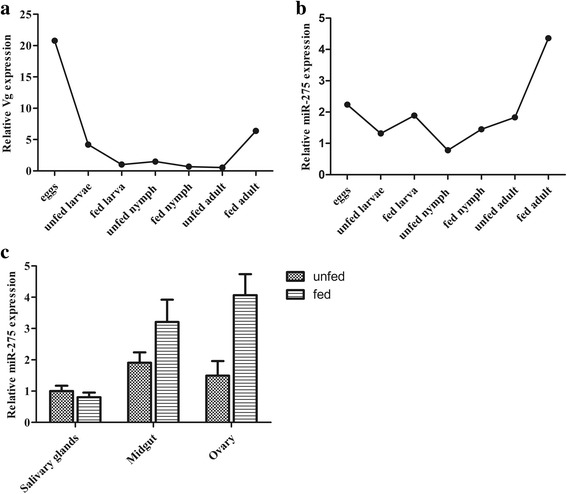
Mature miR-275 and Vg-2 expression in different developmental stages and tissues. **a** Relative expression of Vg-2 was analyzed in eggs, unfed larvae, fed larvae, unfed nymphs, fed nymphs, unfed adults and fed adults. **b** Relative expression of miR-275 was analyzed in eggs, unfed larvae, fed larvae, unfed nymphs, fed nymphs, unfed adults and fed adults. **c** Relative expression of miR-275 in salivary glands, midgut and ovary in unfed and fed female ticks. Data represent three biological replicates with three technical replicates and are shown as the mean ± SEM

To investigate the tissue- and developmental stage-specific expression of miR-275 in ticks, we measured expression levels of mature miR-275 at different developmental stages (egg, unfed larva, fed larva, unfed nymph, fed nymph, unfed adult and fed adult) and in various tissues (midgut, ovary, and salivary glands) from unfed and fed female ticks using real-time PCR. Expression analysis of mature miR-275 in different developmental stages showed that the expression level of miR-275 peaked at the fed adult stage (Fig. 2b). The expression of Vg-2 was highest at the egg stage and then drastically declined in unfed larvae (Fig. 2a). The miR-275 and Vg-2 levels increased in fed adults (Fig. 2a, b). Analysis of different tissues showed that mature miR-275 levels were higher in midgut than those of other studied tissues in unfed female ticks, while levels were the highest in the ovary of fed female ticks (Fig. 2c).

### Inhibition of miR-275 severely affects blood digestion and ovary development

**Fig. 3:**
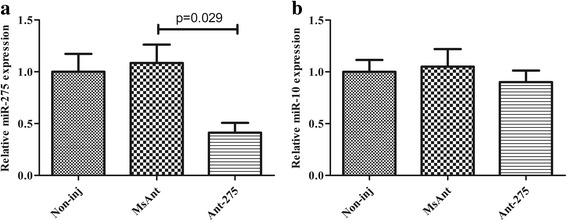
Mature miR-275 is inhibited by the miR-275 antagomir. **a** Relative mature miR-275 expression in fed female ticks. **b** Relative mature miR-10 expression in fed female ticks. Data represent three biological replicates with three technical replicates and are shown as the mean ± SEM

To further investigate the potential function of miR-275 in adult female ticks, we silenced miR-275 using Ant-275. Each unfed female tick was microinjected with Ant-275 or MsAnt. Real-time PCR was performed to assess the silencing efficiency of Ant-275 in fed adults. The results showed that miR-275 expression levels decreased to 41.3% after injection of Ant-275 compared with those of MsAnt and non-injection controls (Fig. 3a) (*t*_(4)_ = 3.336, *P* = 0.029). The expression level of other miRNAs, such as miR-10, was not significantly changed after injection of Ant-275 (Fig. 3b).

**Fig. 4:**
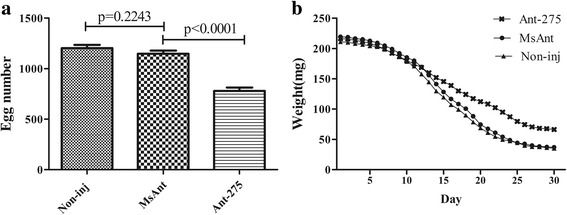
miR-275 inhibition affects blood digestion. **a** Egg numbers of female ticks injected with Ant-275 or MsAnt or non-injected controls. **b** Weight change of fed female ticks injected with Ant-275 or MsAnt or non-injected controls. **a**-**b** Data represent three biological replicates with three technical replicates and are shown as the mean ± SEM

**Fig. 5:**
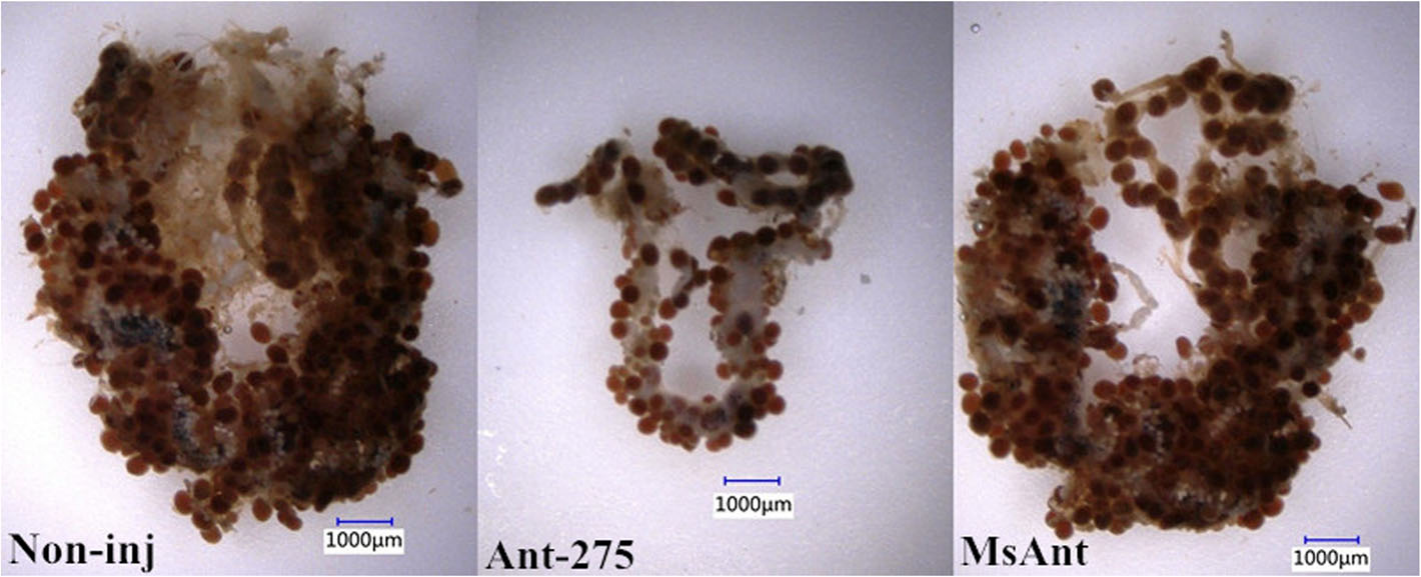
miR-275 inhibition affects ovary development. Ovaries isolated from fed female ticks injected with Ant-275 and non-injected controls

Phenotypic manifestations were also monitored in the female ticks after treatment with Ant-275. There was no difference in blood-feeding between Ant-275-treated and MsAnt control ticks. However, egg deposition was significantly reduced after Ant-275 treatment. The average number of eggs from Ant-275-treated female ticks was 779, whereas that from non-injection control and negative control females was 1203 and 1148, respectively (t_(58)_ = 8.173, *P* < 0.0001) (Fig. 4a). Analysis of the weight change of fed females showed that compared to the control groups, fed female ticks injected with Ant-275 showed much slower decreases in body weight. Six days after engorgement, most *H. longicornis* females began to lay eggs and lost weight, but the weight of those injected with Ant-275 decreased very slowly (Fig. 4b). The weight of Ant-275 background females was 66.4 ± 1.785 mg, but the MsAnt- and non-injection groups weighed 37.00 ± 0.875 mg and 35.23 ± 1.003 mg on the 30th day after engorgement. The average duration of egg laying was 28.10 ± 0.246 days for Ant-275-injected females and 25.33 ± 0.226 and 26.30 ± 0.329 days for non-injected and MsAnt-injected females, respectively. In addition, the rate of weight decrease of engorged females was 69.33 ± 1.547% for the Ant-275 group and 83.94 ± 0.913% and 82.69 ± 0.943% for the non- injection and MsAnt groups, respectively (Table 3). Furthermore, ovarian development of the fed female ticks was observed after they began to lay eggs. Compared to non-injection controls and MsAnt controls, ovaries collecting from fed female ticks treated with Ant-275 were dramatically smaller, and the number of developing oocytes was significantly decreased (Fig. 5).

**Table 3:** The effect of Ant-275 on blood digestion of *Haemaphysalis* longicornis. Data are shown as the mean ± SEM

Group	Change rate of engorged weight (%)^a^	Duration of laying eggs (days)	Final weight (mg)^b^
Ant-275	69.33 ± 1.547^*^	28.10 ± 0.246*	66.40 ± 1.785***
MsAnt	82.69 ± 0.943	26.30 ± 0.329	37.00 ± 0.875
Non-inj	83.94 ± 0.913	25.33 ± 0.226	35.23 ± 1.003

^a^The percentage of change rate of engorged weight was calculated as (engorged weight-final weight)/engorged weight

^b^Final weight was the weight on the 30^th^ day after engorgement (female ticks stop laying and die)

*P < 0.05; ***P < 0.0001 (Student’s *t-*test)

### Phenotype rescue experiment

**Fig. 6:**
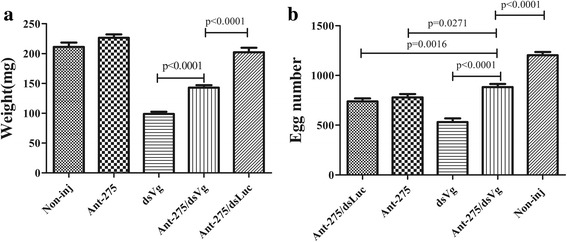
Vg-2 RNAi rescues miR-275 inhibition phenotypes. **a** Body weight after blood meal. **b** Number of eggs deposited after blood meal of ticks injected with Ant-275 or dsVg and co-injected with Ant-275 and dsVg or Ant-275 and dsLuc. Data represent three biological replicates with three technical replicates and are shown as the mean ± SEM

To further confirm Vg-2 as an authentic miR-275 target gene *in vivo*, we conducted phenotype rescue experiment using Vg-2 RNAi of female ticks with an Ant-275 background. We expected that the RNAi-mediated knockdown of the physiologically relevant target of miR-275 would alleviate the adverse phenotypes caused by miR-275 depletion. Co-injection of Ant-275/dsRNA could partially alleviate these phenotypes. The Ant-275/dsRNA female tick body weight significantly increased after a blood meal compared with that of dsVg ticks (*t*_(58)_ = 8.048, *P* < 0.0001) (Fig. 6a), and egg deposition was greater than that of dsVg ticks (*t*_(58)_ = 7.167, *P* < 0.0001) but still lower than that of the non-injection group (*t*_(58)_ = 7.082, *P* < 0.0001) (Fig. 6b). Thus, the results indicated that Vg-2 is an authentic target of miR-275 *in vivo*.

### Vg-2 RNAi

**Fig. 7:**
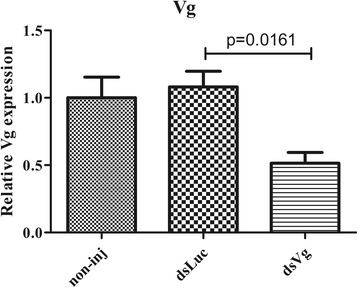
Vg-2 is inhibited by dsRNA. The expression of Vg-2 after injection of dsRNA. Data represent three biological replicates with three technical replicates and are shown as the mean ± SEM

Vg-2 expression was very low in unfed ticks and then increased after blood-feeding. Therefore, to assess the silencing efficiency of Vg dsRNA, we performed real-time PCR to measure Vg-2 after the blood meal. The results showed that Vg-2 expression decreased not only compared to that of non-injected ticks but also that of the dsLuc-injected controls (*t*_(4)_ = 3.999, *P* = 0.0161) (Fig. 7). RNAi silencing of Vg-2 substantially inhibited blood-feeding and egg laying of female ticks. The engorged weight was 98.77 ± 2.268 mg for the dsVg group, while it was 211.20 ± 3.593 mg and 198.68 ± 6.880 mg for the non-injection and dsLuc groups, respectively, the engorged weight was significantly decreased (t_(58)_ = 13.79, *P* < 0.0001). The percentage of egg weight/body weight was 25.9 ± 0.9572% for the dsVg group but 58.6 ± 1.986% and 54.1 ± 1.559% for the non-injection and dsLuc groups, respectively, the percentage of egg weight/body weight treatment with dsVg was significantly decreased (t_(58)_ = 15.42, *P* < 0.0001) (Table 4).

**Table 4:** Effect of Vg-2 dsRNA treatment. Data are shown as the mean ± SEM

Group	Engorged weight (mg)	Egg weight/Body weight (%)
Vg dsRNA	98.77 ± 2.268***	25.9 ± 0.9572***
Luciferase dsRNA	198.68 ± 6.880	54.1 ± 1.559
Non-injection	211.20 ± 3.593	58.6 ± 1.986

***P < 0.0001 (Student’s *t-*test)

## Discussion

Vgs have many miR-275 binding sites, but only Vg-2 had two binding sites with a strong 7-mer seed match site at positions 2–8 for miR-275. A previous study showed that Vg-2 was detected in the ovary and is crucial for ovarian development; therefore, we chose these two canonical seed binding sites for further study. A luciferase reporter vector co-transfected with miR-275 mimic drastically inhibited the *Renilla* luciferase activity, which demonstrated that Vg-2 is a miR-275 target *in vitro*. Furthermore, Vg-2 RNAi with Ant-275 partially alleviated egg deposition and blood-feeding and has been successfully used in rescuing miRNA mutant phenotypes in mosquitoes and *Drosophila* [24, 25]. Thus, the Dual-Luciferase reporter assay system and RNAi demonstrated *in vitro* and *in vivo* that Vg-2 is an authentic target of miR-275 in *H. longicornis*. This is the first report to use the Dual-Luciferase reporter system to verify microRNAs and their target genes in ticks.

Shiping Liu et al. [24] showed that injection of Ant-1174 significantly decreased the egg deposition, but co-injection of Ant-1174/dsSHMT partially reduced the egg deposition, which demonstrated that SHMT (serine hydroxymethyltransferase) was a target gene of miRNA-1174 *in vivo*. In this study, co-injection of dsVg and Ant-275 partially recovered the phenotype; miR-275 was inhibited and did not bind to Vg, and therefore, Vg was only inhibited by dsRNA. These results are similar to those of a previous study.

In a previous study, miR-275 was shown to play an important role in egg development and blood digestion in the mosquito *Aedes aegypti* [21]. Similarly, our study was first to demonstrate that miR-275 and its target gene Vg-2 also regulated blood digestion and ovary development in ticks. Inhibition of *H. longicornis* miR-275 weakened the capability for blood digestion and had severe effects on ovarian development and egg deposition. Ovaries were substantially smaller after inhibition of miR-275, which implied that decreased egg deposition may be attributed to the failed development of the ovaries. Similar to a previous study [15], we found that the egg number and the engorged weight were significantly reduced with Vg-2 RNAi. Because the expression of Vg was very low in unfed ticks (increased after feeding) and barely detected by real-time PCR, we assessed the efficiency of Vg-2 RNAi after feeding and found that it was 51.4%, which satisfied the experimental requirements.

Expression analysis of miR-275 and Vg-2 in different developmental stage of ticks showed that miR-275 and Vg-2 increased in the fed adult stage, suggesting that miR-275 regulates Vg-2 at the fed adult stage. The miR-275 tissue-specific results indicated high levels of miR-275 in unfed tick midgut, and expression was upregulated 2.72-fold in the ovary and 1.68-fold in the midgut after blood-feeding. The results suggested that miR-275 is predominantly involved in ovary development and blood digestion, further demonstrating that miR-275 has an important role in egg development. In addition, approximately 6 days after blood-feeding, most *H. longicornis* females began to lay eggs and gradually lost weight. However, the weight of female ticks with Ant-275 treatment decreased very slowly, the egg laying period was prolonged approximately 2 days, and the final weight was heavier than that of the control groups. These abnormal results suggest that the blood was not digested entirely in the midgut; depletion of miR-275 slowed blood digestion. The blood meal provides a variety of nutrients for ovary development and egg laying; thus, arrested ovary development and lower egg number may be caused by digestive disorders. Therefore, blood digestion and ovary development are inseparable. However, blood intake of female ticks was not affected, suggesting that miR-275 carries out its functions after blood meal. miR-275 has been found in multiple arthropods [21], especially hematophagous species. Therefore, this widespread phenomenon of miR-275 may be essential for growth and development in arthropods.

In mosquitoes, several specific miRNA-regulated blood digestion and egg development processes have been identified [24, 26], but there is little research about miRNAs in ticks. Both miR-5316b and miR-5331 are novel miRNAs in eggs, suggesting that these two miRNAs are indispensable for egg development. miR-307, miR-5317 and miR-5318 were only detected in female ticks, indicating they may be involved in reproduction [20]. This work is the first to elucidate the function of miRNAs in *H. longicornis* ticks. These results provide reliable information for further analyses of egg development and blood digestion in ticks. In this work, we showed that miR-275 targeted Vg-2 and regulated ovary development and blood digestion, indicating that miR-275 has similar functionality in *H. longicornis* and mosquito; thus, specific miRNAs are important to hematophagous arthropods and may have similar functions. Therefore, our study may provide a new way to control the reproduction of ticks.

## Conclusion

To our knowledge, this is the first report showing that miR-275 targets Vg-2 and regulates ovary development and blood digestion in *H. longicornis.* Our data suggest that miR-275 and Vg-2 are essential for reproduction and blood digestion of female ticks. Thus, these results suggest new methods for researching the development and reproduction of ticks.
